# Piperlongumine Alleviates Mouse Colitis and Colitis-Associated Colorectal Cancer

**DOI:** 10.3389/fphar.2020.586885

**Published:** 2020-11-12

**Authors:** Jia-Rong Huang, Sheng-Te Wang, Meng-Ning Wei, Kun Liu, Jing-Wen Fu, Zi-Hao Xing, Zhi Shi

**Affiliations:** ^1^ Department of Cell Biology, Institute of Biomedicine, National Engineering Research Center of Genetic Medicine, MOE Key Laboratory of Tumor Molecular Biology, Guangdong Provincial Key Laboratory of Bioengineering Medicine, College of Life Science and Technology, Jinan University, Guangzhou, China; ^2^ Affiliated High School of South China Normal University, Guangzhou, China

**Keywords:** piperlongumine, Colitis, colorectal cancer

## Abstract

Colorectal cancer is one of the most common and lethal cancers in the world. An important causative factor of colorectal cancer is ulcerative colitis. In this study, we investigated the therapeutic effects of piperlongumine (PL) on the dextran sulfate sodium (DSS)-induced acute colitis and azoxymethane (AOM)/DSS-induced colorectal cancer mouse models. Our results showed that PL could inhibit the inflammation of DSS-induced mouse colitis and reduce the number of large neoplasms (diameter >2 mm) of AOM/DSS-induced mouse colorectal cancer by downregulation of proinflammatory cytokines cyclooxygenase-2 and interleukin-6 and epithelial-mesenchymal transition-related factors, β-catenin, and snail expressions, but fail to improve the colitis symptoms and to decrease the incidence of colonic neoplasms and the number of small neoplasms (diameter <2 mm). These data suggested that PL might be an effective agent in treating colitis and colorectal cancer.

## Introduction

Colorectal cancer including cancer of the colon or rectum is one of the most common and lethal cancers in the world (Siegel et al., 2020). Although the pathogenesis of colon cancer is unclear, an important causative factor of colorectal cancer is ulcerative colitis (Rogler, 2014). Ulcerative colitis is a chronic and idiopathic inflammatory bowel disease characterized by relapsing and remitting mucosal inflammation of the rectum and colon (Kulaylat and Dayton, 2010). Multiple factors are involved in cancer-related inflammation such as cyclooxygenase-2 (COX-2) and interleukin-6 (IL-6), and are also important for the epithelial–mesenchymal transition (EMT), which is a process whereby epithelial cells are decreased adhesion and enhanced migration or invasion to initiate cancer invasion and metastasis (Cao et al., 2015; West et al., 2015). Therefore, suppression of inflammation is a potential strategy to treat ulcerative colitis and colorectal cancer.

Piperlongumine (PL) isolated from the long pepper (*Piperlongum L.*) is an active alkaloid and exhibits a broad spectrum of biological activities including antiangiogenic, antiatherosclerotic, antibacterial, anti-inflammation, and antitumor, etc (Bezerra et al., 2013; Tripathi and Biswal, 2020). Our previous studies have demonstrated that PL can inhibit cell proliferation and induce cell apoptosis in human ovarian cancer and glioblastoma multiforme cells (Liu et al., 2013; Gong et al., 2014; Nan et al., 2019). In this study, we investigated the therapeutic effects of PL on the DSS-induced acute colitis and AOM/DSS-induced colorectal cancer mouse models.

## Materials and Methods

### Reagents

DSS (#60316ES76) was purchased from Yeasen Biotech. AOM (#A5486) was ordered from Sigma-Aldrich. PL (#A4455) was purchased from APExBIO. Anti-IL-6 antibody (#4ab080344) was purchased from 4A Biotech. Anti-COX-2 antibody (#BA0738) was purchased from Boster Biotech. Anti-β-catenin antibody (#610154) was ordered from BD Biosciences. Anti-snail antibody (#RLT4351) was purchased from Ruiying Biological. Anti-β-tubulin antibody (#KM9003T) was purchased from Sungene Biotech.

### Mice Models of Dextran Sulfate Sodium-Induced Acute Colitis and Acute Colitis and Azoxymethane/Dextran Sulfate Sodium-Induced Colorectal Cancer

**FIGURE 1:**
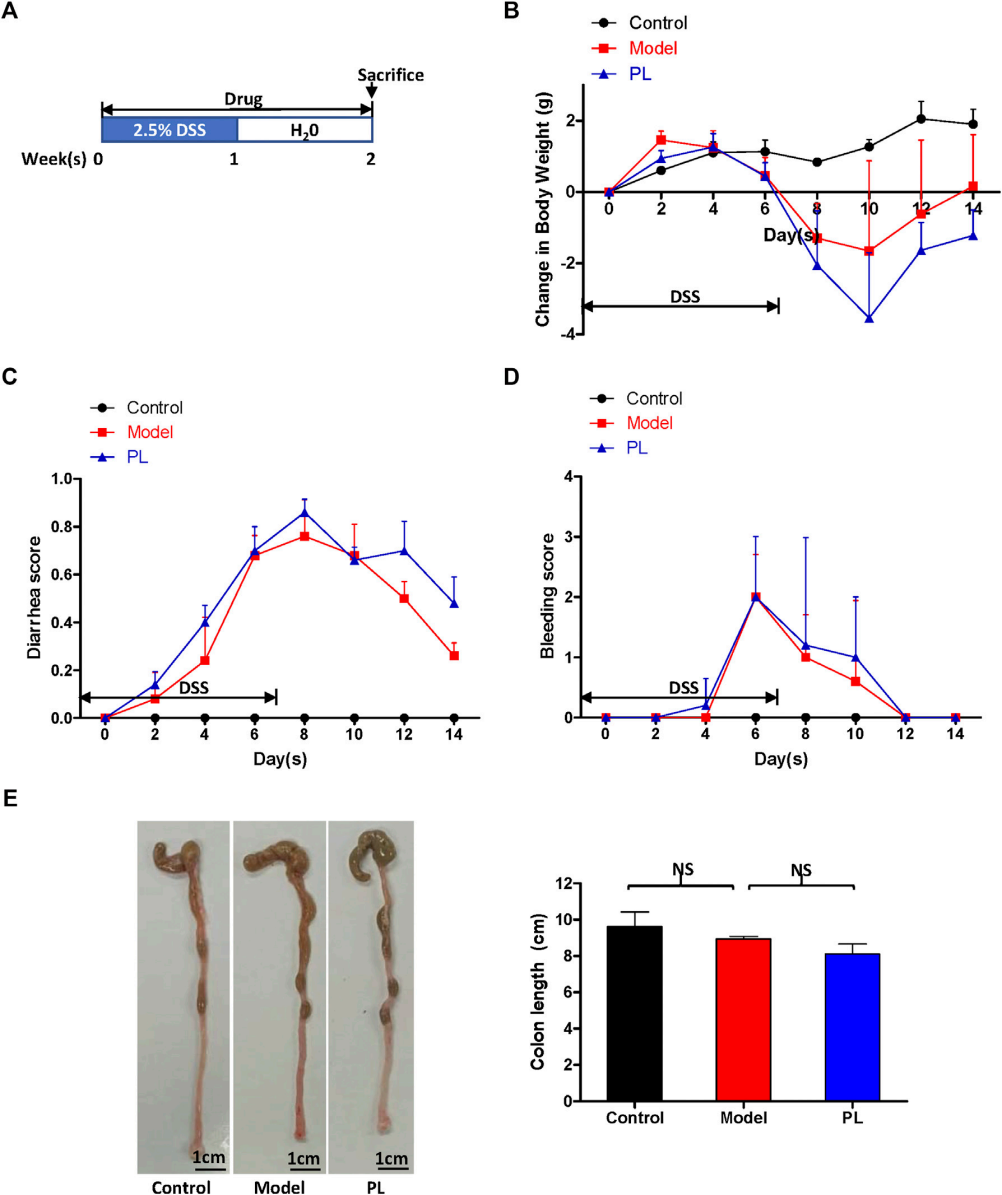
Piperlongumine (PL) fails to improve the symptoms and to alert the colon length in dextran sulfate sodium (DSS)-induced acute colitis mouse models **(A)** The schematic diagram of DSS-induced colitis. The change in body weight of mice **(B)**, diarrhea score **(C)**, bleeding score **(D)**, and the representative whole colons and colon length **(E)** are shown. Note: Control, no treated group; model, DSS-treated group; PL, DSS in combination with PL-treated group. NS, not significant.

**FIGURE 2:**
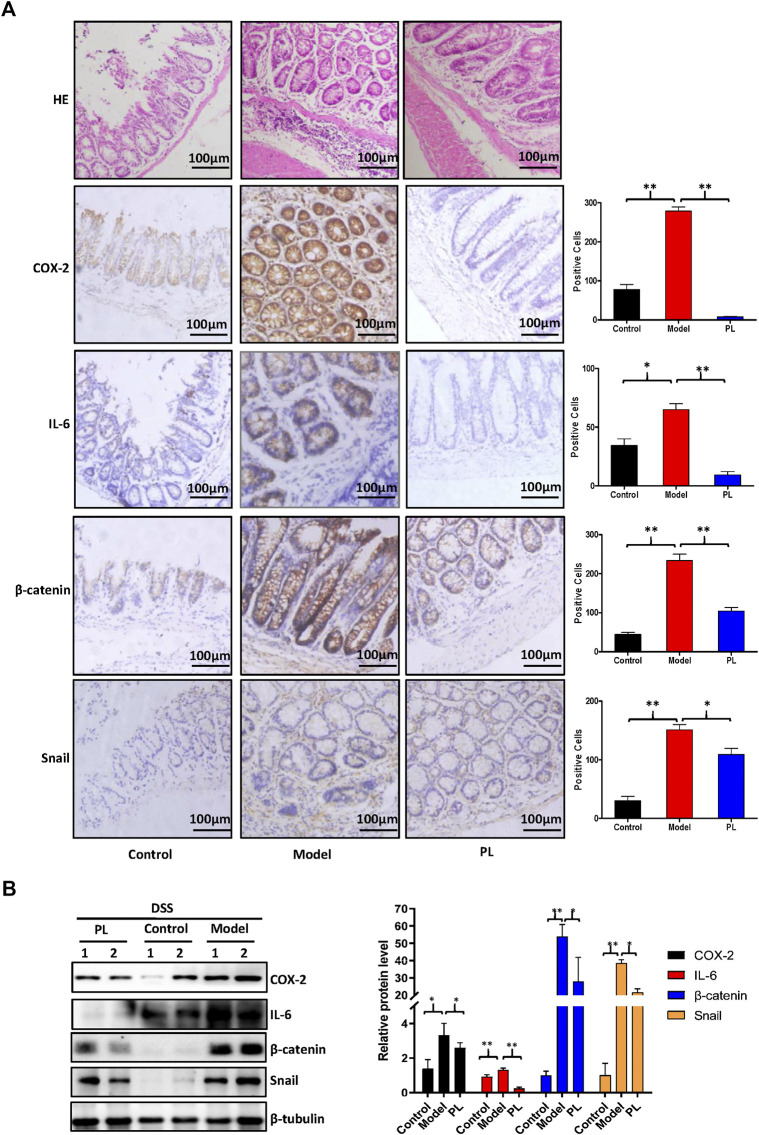
Piperlongumine (PL) inhibits the protein expressions of cyclooxygenase-2 (COX-2), interleukin-6 (IL-6), β-catenin, and snail in dextran sulfate sodium (DSS)-induced acute colitis mouse models. **(A)** Colon sections were stained with H&E and the indicated antibodies. **(B)** The protein expression was examined by Western blot with the indicated antibodies after lysing colon tissues from two mice of each group, and β-tubulin was used as loading control. The representative results and quantified data are shown. Note: Control, no treated group; model, DSS-treated group; PL, DSS in combination with PL-treated group. *p < 0.05, and **p < 0.01 vs. corresponding group.

Seven-week-old male BALB/c mice were obtained from the Guangdong Medical Laboratory Animal Center. Five mice were randomized into each group. Acute colitis was induced in mice by providing drinking water with 2.5% DSS for 1 week, followed by switching to regular drinking water for another 1 week. Mice were injected with PL (10 mg/kg) or solution control (0.5% methylcellulose) intraperitoneally every day for 2 weeks (Figure 1A). Colorectal cancer was induced in mice by intraperitoneal injection of AOM (10 mg/kg), while the mice were maintained with regular drinking water for 1 week and then subjecting the mice to three cycles of DSS treatment, with each cycle including 2.5% DSS treatment for 1 week and regular water for 2 weeks. Mice were injected with PL (10 mg/kg) or solution control (0.5% methylcellulose) intraperitoneally every day for 9 weeks (Figure 2A) (Wei et al., 2016).

### Clinical Assessment of Dextran Sulfate Sodium-Induced Acute Colitis and Acute Colitis and Azoxymethane/Dextran Sulfate Sodium-Induced Colorectal Cancer

Body weight, diarrhea degree, and bleeding degree in the rectum were determined in mice every two days. Diarrhea degree was scored 0 for well-formed pellets, 0.3 for soft pellets, 0.6 for pasty stools, 0.9 for liquid stools. Bleeding degree was scored as 0, when there was no blood; 2, for slight bleeding; 4, for gross bleeding (Wei et al., 2016).

### Hematoxylin and Eosin and Immunohistochemistry Staining

Formalin-fixed paraffin-embedded tissue slides were deparaffinized using xylene and graded ethyl alcohol and then stained with haematoxylin and eosin (H&E) solution (Supplementary Figure S1). For immunohistochemistry staining, antigen retrieval was performed by boiling the slides in 0.01 M citrate buffer (pH = 6) in a microwave oven for 10 min and cooling at room temperature. The slides were then incubated with 0.05% Triton X-100 in PBS for 5 min, followed by sequential treatment in a humidified chamber after quenching endogenous peroxides with 3% H_2_O_2_ in MeOH, blocking serum with avidin for 20 min, first antibody overnight at 4°C, secondary antibody for 20 min, hydrogen peroxidase for 15 min, and peroxidase substrate solution for 20 min at room temperature. The stained slides were then counterstained with hematoxylin and cover slipped. The following formula was used to quantify protein expression: immunohistochemical score = percentage of positive cells × intensity score. The intensity was scored as follows: 0, negative; 1, weak; 2, moderate; and 3, intense. An immunohistochemical score of ≥50 was considered as positive (Zhang et al., 2017).

### Western Blot

Shredded colon tissues were harvested and washed twice with cold PBS, then resuspended and lysed in RIPA buffer (1% NP-40, 0.5% sodium deoxycholate,0.1% SDS, 10 ng/ml PMSF, 0.03% aprotinin, 1 *μ*M sodium orthovanadate) at 4°C for 30 min. Lysates were centrifuged for 10 min at 14,000 × g and supernatants were stored at −80°C as whole cell extracts. Total protein concentrations were determined with Bradford assay. Thirty μg proteins of each sample were separated on 12% SDS-PAGE gels and transferred to polyvinylidene difluoride membranes. Membranes were blocked with 5% BSA and incubated with the indicated primary antibodies. Corresponding horseradish peroxidase-conjugated secondary antibodies were used against each primary antibody. Proteins were detected using the chemiluminescent detection reagents and Bio-Rad image system. The semiquantitative analysis of protein bands was carried out by software ImageJ (Yuan et al., 2018).

### Statistical Analysis

A student’s t-test was used to compare individual data points among each group. A value of *p* < 0.05 was considered to indicate a significant difference between groups.

## Results

### Piperlongumine Inhibits the Inflammation in Dextran Sulfate Sodium-Induced Acute Colitis Mouse Models

DSS-induced acute colitis mouse model was generated by providing drinking water with 2.5% DSS for 1 week, followed by switching to regular drinking water for another 1 week to evaluate the anti-inflammation efficacy of PL (Figure 1A **)**. As shown in Figures 1B–E, DSS-treated mice exhibited the colitis symptoms (body weight loss, diarrhea, and rectal bleeding), and PL administration failed to improve these colitis symptoms and to alert the colon length. Histopathologically, DSS-treated mice showed epithelial architecture destruction with loss of crypts and epithelial integrity and inflammatory cell infiltration, and PL administration significantly reduced inflammatory cell infiltration in the mucosa (Figure 2A **)**. Moreover, both results of immunohistochemistry staining and Western blot showed that elevated protein expressions of COX-2, IL-6, β-catenin, and snail were detected in the epithelial cells of DSS-treated mice, and these increases were prevented by PL (Figures 2A,B **)**.

### Piperlongumine Attenuates the Tumor Growth in Acute Colitis and Azoxymethane/Dextran Sulfate Sodium-Induced Colorectal Cancer Mouse Models

**FIGURE 3:**
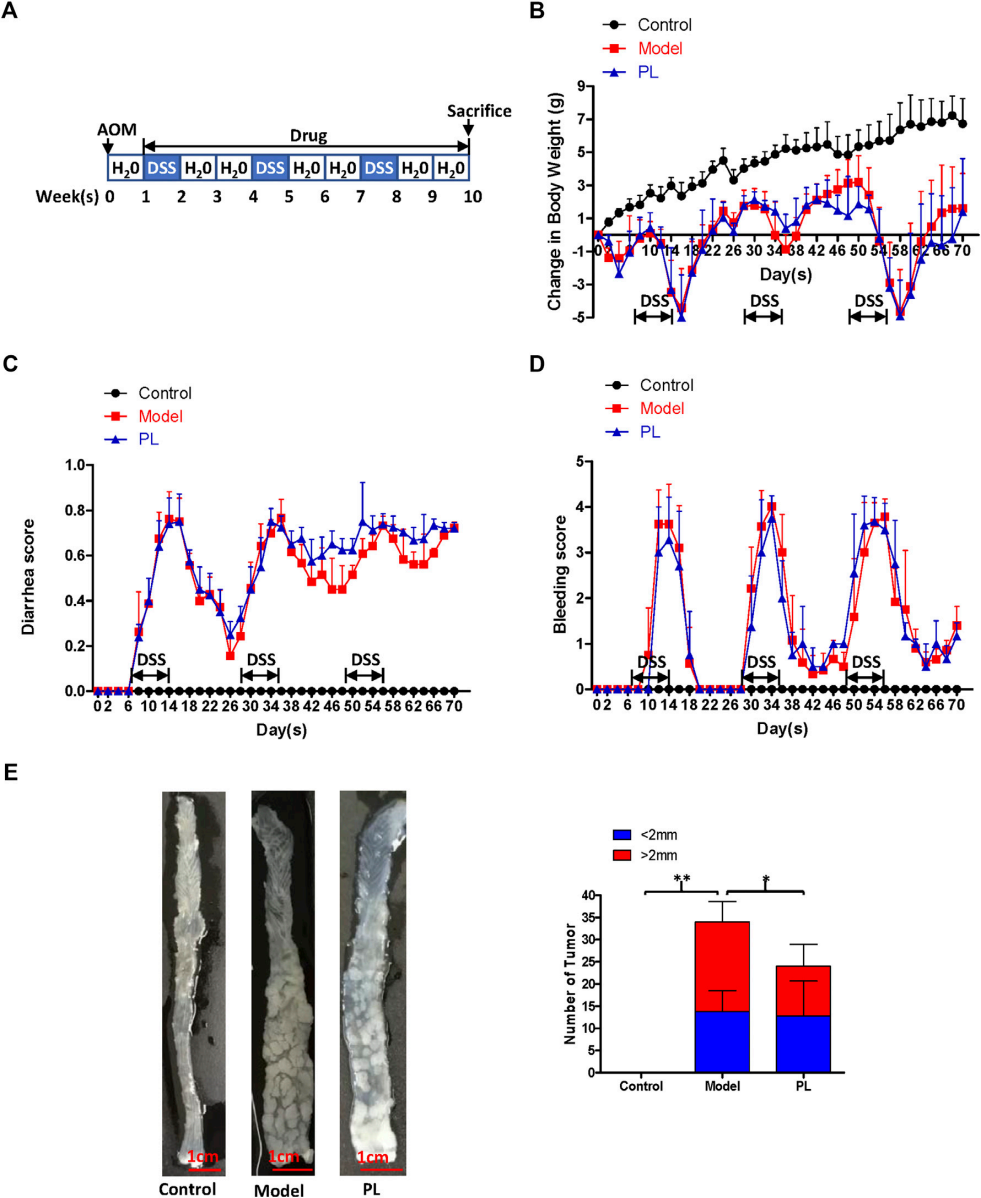
Piperlongumine (PL) fails to improve the symptoms, but inhibited the tumor growth in acute colitis and azoxymethane (AOM)/dextran sulfate sodium (DSS)-induced colorectal cancer mouse models **(A)** The schematic diagram of AOM/DSS-induced colorectal cancer. The change in body weight of mice **(B)**, diarrhea score **(C)**, bleeding score **(D)**, and the representative whole colons and number of colon cancer **(E)** are shown. Note: Control, no treated group; model, AOM/DSS-treated group; PL, AOM/DSS in combination with PL-treated group. *p < 0.05, and **p < 0.01 vs. corresponding group.

**FIGURE 4:**
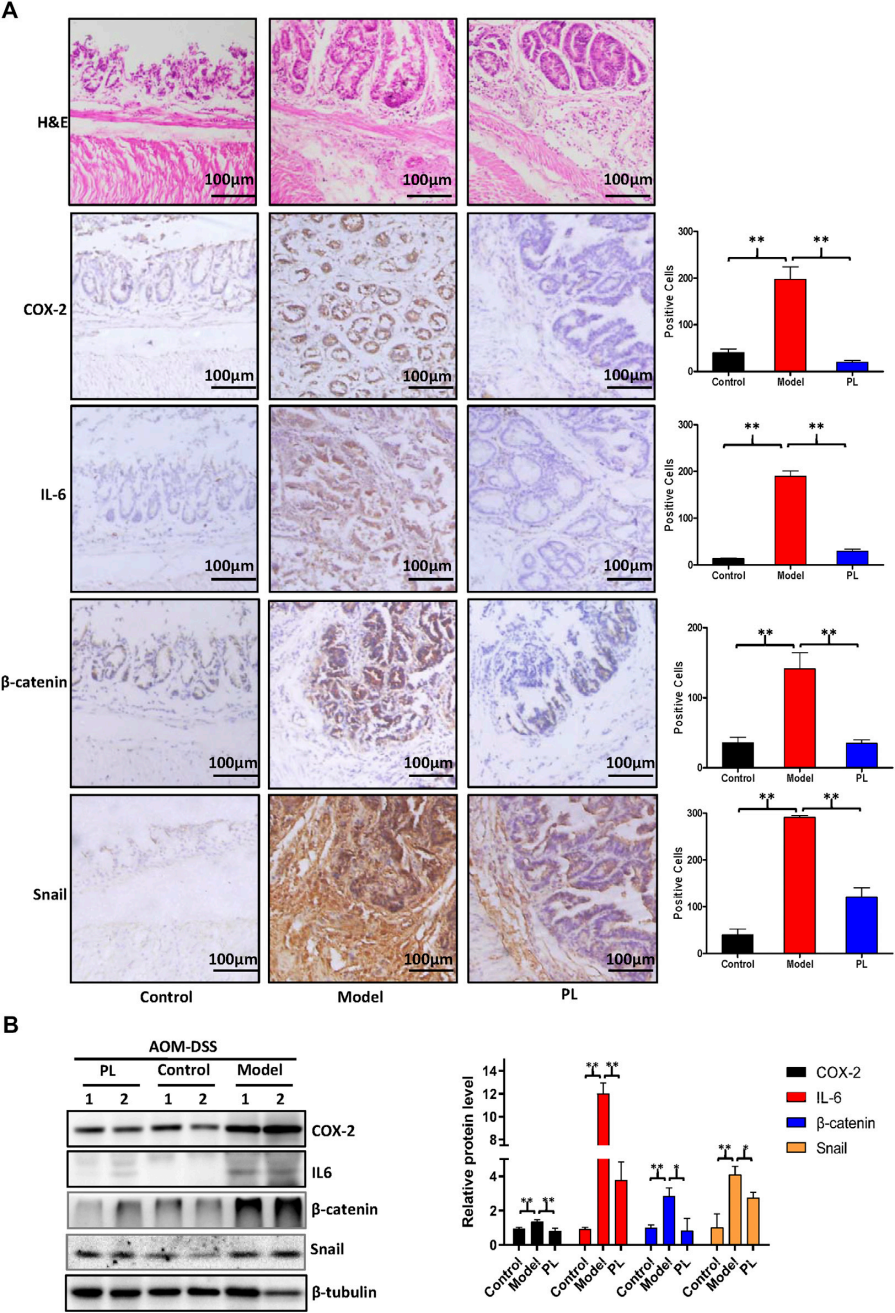
Piperlongumine (PL) inhibits the protein expressions of cyclooxygenase-2 (COX-2), interleukin-6 (IL-6), β-catenin, and snail in acute colitis and azoxymethane (AOM)/DSS-induced colorectal cancer mouse models **(A)** Colon sections were stained with H&E and the indicated antibodies **(B)** The protein expression was examined by Western blot with the indicated antibodies after lysing colon tissues from two mice of each group, and β-tubulin was used as loading control. The representative results and quantified data are shown. Note: Control, no treated group; model, AOM/DSS-treated group; PL, AOM/DSS in combination with PL-treated group. *p < 0.05, and **p < 0.01 vs. corresponding group.

As PL could inhibit the inflammation in DSS-induced acute colitis in mice, the therapeutic effect of PL was further evaluated in AOM/DSS-induced colorectal cancer mouse models generated by intraperitoneal injection of AOM (10 mg/kg), while the mice were maintained with regular drinking water for 1 week and then subjecting the mice to three cycles of DSS treatment, with each cycle including 2.5% DSS treatment for 1 week and regular water for 2 weeks (Figure 3A **)**. As show in Figures 3B–D, symptomatic parameters (body weight loss, diarrhea, and rectal bleeding) were observed after DSS administration and decreased after DSS withdrawal in AOM/DSS-treated mice, and PL administration failed to improve these symptoms. AOM/DSS treatment resulted in 100% incidence of colonic neoplasms in mice of all groups. PL administration failed to decrease the incidence of colonic neoplasms and the number of small neoplasms (diameter <2 mm), but significantly reduced the number of large neoplasms (diameter >2 mm) (Figure 3E). The results of H&E staining exhibited colon adenocarcinomas with dysplasia in AOM/DSS-treated mice (Figures 4A). Furthermore, both results of immunohistochemistry staining and Western blot showed that elevated protein expressions of COX-2, IL-6, β-catenin, and snail were also detected in the tumor cells of AOM/DSS-treated mice, and these increases were prevented by PL (Figures 4A,B **)**.

## Discussion

The inflammation–dysplasia–carcinoma is a common development process of colorectal cancer (Lichtenstern et al., 2020). DSS-induced mouse colitis has been demonstrated to be similar to human colitis, and AOM/DSS-induced mouse colorectal cancer has been proven to rapidly mimic the abnormal crypt foci–adenoma–carcinoma sequence of human colorectal cancer (Neurath, 2012). Therefore, the mouse models of DSS-induced colitis and AOM/DSS-induced colorectal cancer have been widely used to explore the pathophysiologic mechanisms and therapeutic strategies of human colitis and colorectal cancer (Snider et al., 2016). In this study, we found that PL could inhibit the inflammation of DSS-induced mouse colitis and reduce the number of large neoplasms (diameter >2 mm) of AOM/DSS-induced mouse colorectal cancer by downregulation of proinflammatory cytokines COX-2 and IL-6 and EMT-related factors β-catenin and snail expressions, but fail to improve the colitis symptoms and to decrease the incidence of colonic neoplasms and the number of small neoplasms (diameter <2 mm). It might be because AOM administration was a week ahead of PL treatment, and PL can affect DSS-induced inflammation, but not AOM-induced mutagenesis and tumorigenesis. Similarly, a recent report has shown that PL could suppress tumor cell growth and proliferation in DMH/DSS-induced experimental colon cancer by targeting Ras/PI3K/Akt/mTOR signaling axis (Kumar and Agnihotri, 2019). PL-induced Cyclin D1 downregulation and tumor suppression in colorectal cancer cells by suppression of ERKs/Akt-mediated c-Fos expression (Gao et al., 2020). PL could also induce apoptosis by activation of JNK in colorectal cancer HCT116 cells independent of Bax, p21 and p53 status (Li et al., 2015; DA Silva Machado et al., 2018). Additionally, PL increased the sensitivity of colorectal cancer cells to oxaliplatin and radiation by inducing reactive oxygen species production (Chen et al., 2019; Wang et al., 2019). However, the application of PL for human colitis and colorectal cancer treatment need to be further investigated.

In conclusion, our results revealed that PL could inhibit the inflammation of DSS-induced mouse colitis and tumor growth of AOM/DSS-induced mouse colorectal cancer, indicating that PL might be an effective agent to treat colitis and colorectal cancer.

## Data Availability Statement

The raw data supporting the conclusions of this article will be made available by the authors, without undue reservation, to any qualified researcher.

## Ethics Statement

The animal study was reviewed and approved by the ethics committee of Jinan University.

## Author Contributions

JH, SW and ZS designed the experiments, performed the experiments, analyzed the data and wrote the paper. MW, KL, JF and ZX performed the experiments and wrote the paper. All authors read and approved the final manuscript.

## Funding

This work was supported by funds from the National Key Research and Development Program of China No. 2017YFA0505104 (ZS), the National Natural Science Foundation of China No. 81772540 (ZS) and the Science and Technology Program of Guangdong No. 2019A050510023 (ZS).

## Conflict of Interest

The authors declare that the research was conducted in the absence of any commercial or financial relationships that could be construed as a potential conflict of interest.

## References

[B1] Bezerra D. P. Pessoa C. De Moraes M. O. Saker-Neto N. Silveira E. R. Costa-Lotufo L. V. (2013). Overview of the therapeutic potential of piplartine (piperlongumine). Eur. J. Pharmaceut. Sci. 48, 453–463. 10.1016/j.ejps.2012.12.003 23238172

[B2] Cao H. Xu E. Liu H. Wan L. Lai M. (2015). Epithelial-mesenchymal transition in colorectal cancer metastasis: a system review. Pathol. Res. Pract. 211, 557–569. 10.1016/j.prp.2015.05.010 26092594

[B3] Chen W. Lian W. Yuan Y. Li M. (2019). The synergistic effects of oxaliplatin and piperlongumine on colorectal cancer are mediated by oxidative stress. Cell Death Dis. 10, 600 10.1038/s41419-019-1824-6 31395855 PMC6687721

[B4] DA Silva Machado F. Munari F. M. Scariot F. J. Echeverrigaray S. Aguzzoli C. Pich C. T. (2018). Piperlongumine induces apoptosis in colorectal cancer HCT 116 cells independent of bax, p21 and p53 status. Anticancer Res. 38, 6231–6236. 10.21873/anticanres.12978 30396942

[B5] Gao F. Zhou L. Li M. Liu W. Yang S. Li W. (2020). Inhibition of ERKs/Akt-Mediated c-Fos expression is required for piperlongumine-induced cyclin D1 downregulation and tumor suppression in colorectal cancer cells. Onco. Targets Ther. 13, 5591–5603. 10.2147/ott.s251295 32606774 PMC7304781

[B6] Gong L. H. Chen X. X. Wang H. Jiang Q. W. Pan S. S. Qiu J. G. (2014). Piperlongumine induces apoptosis and synergizes with cisplatin or paclitaxel in human ovarian cancer cells. Oxid. Med. Cell Longev. 2014, 906804 10.1155/2014/906804 24895529 PMC4034765

[B7] Kulaylat M. N. Dayton M. T. (2010). Ulcerative colitis and cancer. J. Surg. Oncol. 101, 706–712. 10.1002/jso.21505 20512947

[B8] Kumar S. Agnihotri N. (2019). Piperlongumine, a piper alkaloid targets Ras/PI3K/Akt/mTOR signaling axis to inhibit tumor cell growth and proliferation in DMH/DSS induced experimental colon cancer. Biomed. Pharmacother. 109, 1462–1477. 10.1016/j.biopha.2018.10.182 30551398

[B9] Li W. Wen C. Bai H. Wang X. Zhang X. Huang L. (2015). JNK signaling pathway is involved in piperlongumine-mediated apoptosis in human colorectal cancer HCT116 cells. Oncol. Lett. 10, 709–715. 10.3892/ol.2015.3371 26622558 PMC4509051

[B10] Lichtenstern C. R. Ngu R. K. Shalapour S. Karin M. (2020). Immunotherapy, inflammation and colorectal cancer. Cells 9, 618 10.3390/cells9030618 PMC7140520 32143413

[B11] Liu J. M. Pan F. Li L. Liu Q. R. Chen Y. Xiong X. X. (2013). Piperlongumine selectively kills glioblastoma multiforme cells via reactive oxygen species accumulation dependent JNK and p38 activation. Biochem. Biophys. Res. Commun. 437, 87–93. 10.1016/j.bbrc.2013.06.042 23796709

[B12] Nan X. W. Gong L. H. Chen X. Zhou H. H. Ye P. P. Yang Y. (2019). Survivin promotes piperlongumine resistance in ovarian cancer. Front. Oncol. 9, 1345 10.3389/fonc.2019.01345 31850227 PMC6895030

[B13] Neurath M. F. (2012). Animal models of inflammatory bowel diseases: illuminating the pathogenesis of colitis, ileitis and cancer. Dig. Dis. 30 (Suppl. 1), 91–94. 10.1159/000341131 23075875

[B14] Rogler G. (2014). Chronic ulcerative colitis and colorectal cancer. Canc. Lett. 345, 235–241. 10.1016/j.canlet.2013.07.032 23941831

[B15] Siegel R. L. Miller K. D. Goding Sauer A. Fedewa S. A. Butterly L. F. Anderson J. C. (2020). Colorectal cancer statistics, 2020. CA A Cancer J. Clin. 70, 145–164. 10.3322/caac.21601 32133645

[B16] Snider A. J. Bialkowska A. B. Ghaleb A. M. Yang V. W. Obeid L. M. Hannun Y. A. (2016). Murine model for colitis-associated cancer of the colon. Methods Mol. Biol. 1438, 245–254. 10.1007/978-1-4939-3661-8_14 27150094 PMC5657253

[B17] Tripathi S. K. Biswal B. K. (2020). Piperlongumine, a potent anticancer phytotherapeutic: perspectives on contemporary status and future possibilities as an anticancer agent. Pharmacol. Res. 156, 104772 10.1016/j.phrs.2020.104772 32283222

[B18] Wang H. Jiang H. Corbet C. De Mey S. Law K. Gevaert T. (2019). Piperlongumine increases sensitivity of colorectal cancer cells to radiation: involvement of ROS production via dual inhibition of glutathione and thioredoxin systems. Canc. Lett. 450, 42–52. 10.1016/j.canlet.2019.02.034 30790679

[B19] Wei T.-T. Lin Y.-T. Tseng R.-Y. Shun C.-T. Lin Y.-C. Wu M.-S. (2016). Prevention of colitis and colitis-associated colorectal cancer by a novel polypharmacological histone deacetylase inhibitor. Clin. Canc. Res. 22 **,** 4158–4169. 10.1158/1078-0432.ccr-15-2379 27528734

[B20] West N. R. Mccuaig S. Franchini F. Powrie F. (2015). Emerging cytokine networks in colorectal cancer. Nat. Rev. Immunol. 15, 615–629. 10.1038/nri3896 26358393

[B21] Yuan M. L. Li P. Xing Z. H. Di J. M. Liu H. Yang A. K. (2018). Inhibition of WEE1 suppresses the tumor growth in laryngeal squamous cell carcinoma. Front. Pharmacol. 9, 1041 10.3389/fphar.2018.01041 30323762 PMC6172786

[B22] Zhang W.-J. Li Y. Wei M.-N. Chen Y. Qiu J.-G. Jiang Q.-W. (2017). Synergistic antitumor activity of regorafenib and lapatinib in preclinical models of human colorectal cancer. Canc. Lett. 386, 100–109. 10.1016/j.canlet.2016.11.011 27864115

