# Supplementary figures and images for: Piperlongumine Alleviates Mouse Colitis and Colitis-Associated Colorectal Cancer
Source: Front Pharmacol. 2020 Nov 12;11:586885. doi: 10.3389/fphar.2020.586885 (PMC7748110; doi:10.3389/fphar.2020.586885)

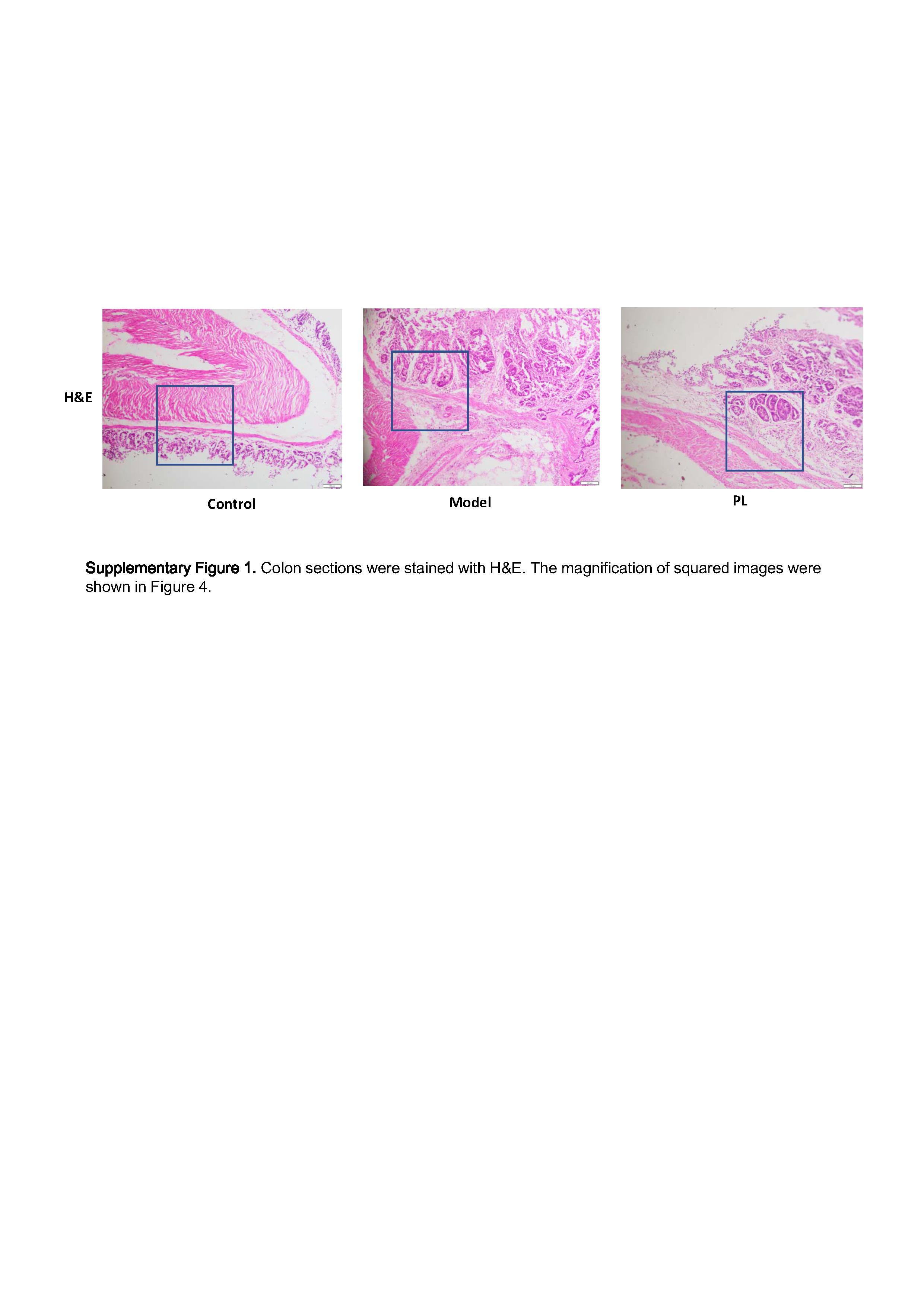

Supplement: Supplementary file 1 [file Image1_v1.TIFF]
